# Restoration of Haemoglobin Level Using Hydrodynamic Gene Therapy with Erythropoietin Does Not Alleviate the Disease Progression in an Anaemic Mouse Model for TGFβ1-Induced Chronic Kidney Disease

**DOI:** 10.1371/journal.pone.0128367

**Published:** 2015-06-05

**Authors:** Lea Pedersen, Lise Wogensen, Niels Marcussen, Claudia R. Cecchi, Trine Dalsgaard, Frederik Dagnæs-Hansen

**Affiliations:** 1 Research Laboratory for Biochemical Pathology, Aarhus University Hospital, Institute of Clinical Medicine, Aarhus, Denmark; 2 Department of Pathology, Odense University Hospital, Odense, Denmark; 3 Department of Biomedicine, Aarhus University, Aarhus, Denmark; Medical University of Graz, AUSTRIA

## Abstract

Erythropoietin, Epo, is a 30.4 kDa glycoprotein hormone produced primarily by the fetal liver and the adult kidney. Epo exerts its haematopoietic effects by stimulating the proliferation and differentiation of erythrocytes with subsequent improved tissue oxygenation. Epo receptors are furthermore expressed in non-haematopoietic tissue and today, Epo is recognised as a cytokine with many pleiotropic effects. We hypothesize that hydrodynamic gene therapy with Epo can restore haemoglobin levels in anaemic transgenic mice and that this will attenuate the extracellular matrix accumulation in the kidneys. The experiment is conducted by hydrodynamic gene transfer of a plasmid encoding murine Epo in a transgenic mouse model that overexpresses TGF-β1 locally in the kidneys. This model develops anaemia due to chronic kidney disease characterised by thickening of the glomerular basement membrane, deposition of mesangial matrix and mild interstitial fibrosis. A group of age matched wildtype littermates are treated accordingly. After a single hydrodynamic administration of plasmid DNA containing murine EPO gene, sustained high haemoglobin levels are observed in both transgenic and wildtype mice from 7.5 ± 0.6 mmol/L to 9.4 ± 1.2 mmol/L and 10.7 ± 0.3 mmol/L to 15.5 ± 0.5 mmol/L, respectively. We did not observe any effects in the thickness of glomerular or tubular basement membrane, on the expression of different collagen types in the kidneys or in kidney function after prolonged treatment with Epo. Thus, Epo treatment in this model of chronic kidney disease normalises haemoglobin levels but has no effect on kidney fibrosis or function.

## Introduction

Patients with various long lasting diseases such as chronic kidney disease, cancer, or acquired immunodeficiency syndrome (AIDS) often develop anaemia due to the underlying disease or as a consequence of treatment [1]. Anaemia is associated with a number of adverse consequences, including cardiovascular mortality and reduced life quality. Erythropoietin, Epo, is a 30.4 kDa glycoprotein hormone produced primarily by the fetal liver and in the adult kidney [2–4]. Renal interstitial cells in mammals are sensitive to hypoxia and respond by producing Epo if the haemoglobin levels are below a certain limit [5,6]. The circulating hormone binds to the Epo-receptor and exerts its hematopoietic effect by stimulating the proliferation and differentiation of erythrocytes with subsequent improved tissue oxygenation. Patients are currently successfully treated with Epo replacement therapy to restore the haemoglobin levels.

Epo-receptors are also expressed in non-haematopoietic tissue and today, Epo is recognised as a hormone with many more pleiotropic effects than previously described [7–9]. Thus, beneficial as well as disadvantageous effects are possible during Epo replacement therapy. Experimental as well as clinical trials have repeatedly demonstrated that treatment of pre-dialysis patients with recombinant human Epo slows the decline in renal function [10]. Many of these studies are, however, limited by short observation periods and are primarily focusing on the effect in acute renal failure. Furthermore, it is unknown whether the restored haemoglobin levels or a direct effect of Epo *per se* are responsible for the different beneficial effects of Epo [10–15].

The effect of prolonged normalisation of haemoglobin levels for the progression of chronic kidney disease needs to be clarified. To address this we have transfected plasmid encoding the murine EPO gene in a transgenic mouse model with chronic kidney disease and anaemia. Our transgenic mouse model, RenTGF-β1, has impaired kidney function due to local overexpression of active porcine TGF-β1 under control of the Renin-1^c^ promotor [16]. The mice demonstrate fibrotic lesions exclusively within the kidneys [17]. When the mice are 2-month-old they show thickening of the glomerular basement membrane (GBM), deposition of mesangial matrix, increased glomerular volume, interstitial fibrosis and proteinuria. The changes are fully developed at 4-months of age.

Hydrodynamic gene therapy is a non-viral method for gene therapy, which diminishes the undesirable immune reactions and off-target effects. This method transfers plasmid DNA in liver cells with subsequent gene expression [18–20]. Mice and rat studies have shown that rapid injection in the tail vein of a large volume buffer containing plasmid DNA, e.g. 8–10% of their bodyweight, leads to high expression from hepatocytes of the plasmid expressed gene [21–24].

The present study was designed to determine whether hydrodynamic gene therapy with Epo could normalise haemoglobin levels in an anaemic transgenic mouse model with chronic kidney disease and to examine the biochemical, functional, and ultrastructural changes in the kidneys after prolonged Epo expression. From this study, we conclude that prolonged Epo expression after a single hydrodynamic Epo plasmid injection restore normal haemoglobin levels in transgenic mice with chronic kidney disease, but treatment with Epo has no effects on kidney morphology and function.

## Methods

### Vectors expressing Epo

The plasmids pUBI-mEPO and pUBI-fLUC (Control) were cloned by PCR amplification of the human ubiquitin C promoter (UBI) from pSBT/UBI-GIN as previously described [25] and ligated into the HindIII restriction site. The murine erythropoietin (Epo) cassette was PCR amplified from PUHD-10.3-mEPO kindly donated by Gehl [26] and inserted after the UBI promoter using the SalI and NotI restriction sites. As control plasmid, we generated the pUBI-fLUC plasmid by replacing the EPO cassette with the firefly luciferase (fLuc) using the same restriction enzymes. The firefly luciferase was PCR amplified from the psi-check 2.2 vector purchased from Promega (Madison, USA). All plasmids were validated by sequencing.

### Animal model and treatment groups

In all experiments we used the previously described RenTGF-β1 mouse strain expressing mutated porcine TGF-β1 under control of the Ren-1^c^ promotor [16]. Two-month-old male RenTGF-β1 transgenic (Tg) mice were divided randomly in four groups. The dose of pUBI-mEPO (Epo) plasmid was decided from previous studies (unpublished data, Frederik Dagnæs-Hansen): 1.25 μg/mouse *n* = 13; 2.5 μg/mouse *n* = 15; or 5 μg/mouse *n* = 5. The dose of the control plasmid pUBI-fLUC (Control) was 5 μg/mouse *n* = 15. Mice that received 5 μg/mouse Epo showed clear signs of polycythemia, including haemoglobin levels above measurement limit and bloodshottend ears and paws, and were terminated. The bodyweights were monitored weekly during the study period and the haemoglobin level was measured from blood taken from the retroorbital plexus using HemoCue 201+ (Abena A/S, Denmark). A group of non-transgenic age- and sex-matched littermates (Wt) were injected with Epo plasmid (1.25 μg/mouse (*n* = 11)), Control plasmid (5 μg/mouse (*n* = 7)) or left untreated (*n* = 8) to examine whether Epo gene expression had adverse effects in normal animals. We chose to treat the Wt mice with the lowest concentration of Epo 1.25 μg/mouse only to avoid severe side effects in the normoxia mice. As reported below, Wt mice treated with Control plasmid had similar body weight, haemoglobin levels, and liver histology compared to untreated mice. Therefore, these mice were pooled in one control group (Wt Control *n =* 15).

All mice were housed in certified animal facilities in a 12-hours dark:light cycle with free accessibility to water and standard chow. At the end of the experiment, all the mice were terminated by exsanguination under anaesthesia with Hypnorm/Dormicum. The animal experiment was specifically approved and performed under guidelines given by the Danish Animal Experiments Inspectorate (2008/561-1512 and 2012-15-2934-00081). EFPIA guideline, “A Good Practice Guide to the Administration of Substances and Removal of Blood, Including Routes and Volumes" [27] was followed carefully throughout the whole study.

### Tail vein injection and collection of material

In order to deliver plasmid to the mice, tail vein injection was performed. Before injection, the mice were weighed and kept under high ambient temperature to dilate the veins. The mice were anaesthetised in a chamber with 3.75% (v/v) isoflurane immediately before injection. Different doses of plasmid DNA contained in sterile Ringer solution (147 mM NaCl, 4 mM KCl, and 1.13 mM CaCl_2_) were administrated in the tail vein in a volume corresponding to 8% of the body weight. The injections were performed within 5–8 seconds and the animals were weighed immediately after injection. Haemostasis at the injection site was observed after applying pressure for 10 seconds. The mice were allowed to recover and the bodyweights were back to baseline level the day after injection. Exclusion criteria for further analyses were prolonged injection time (> 8 seconds) and lack of rise in the haemoglobin levels in the Epo-treated mice. From a subpopulation of mice, 24-hours urine was collected in metabolism cages at the end of the experiment. All the mice were terminated by exsanguination under anaesthesia. One kidney was sampled and immersion fixed for stereology in ¾ Tyrode’s buffer containing 1% glutaraldehyde and 3% paraformaldehyde and one was frozen in liquid nitrogen and stored at -80°C for protein or RNA analysis. A small liver sample (5 mm x 5 mm) was collected and frozen in liquid nitrogen. Another liver and spleen piece was removed (*n* = 4 from all groups), fixed in 4% paraformaldehyde, and embedded in paraffin.

### Histology

To visualise possible adverse morphological effects of the hydrodynamic gene therapy treatment on liver tissue and to identify collagen deposits in the kidneys, paraffin sections were stained by Hematoxylin eosin, Mallory’s trichrome staining for collagen, and Silver staining for reticular fibers and collagen α1(III).

### In vivo bioluminescence imaging

The Control plasmid encodes the luciferase gene, which visualises the anatomic location of plasmid expression. Mice treated with Control plasmid were analysed for bioluminescence 8 days after plasmid administration (Tg *n* = 8, Wt *n* = 5). Briefly, the mice were injected subcutaneously with the substrate D-luciferin potassium salt (Synchem OHG, Felsberg-Altenburg, Germany) at a dose of 150 mg/kg and subsequently anaesthetised with 3.75% isofluorane (Forene, Abbott Scandinavia AB, Solna, Sweden). Anaesthesia was maintained at 2% isofluorane during the bioluminescence scan in the IVIS Spectrum imaging system (Caliper Life Sciences, Hopkinton, MA, USA). The regions of interest from displayed images were quantified as photons/second using the Living Image software 4.2 (Xenogen, Alameda, CA). Presence of a strong signal from the liver region was considered to be an indication of plasmid expression in the liver. Scanning Control mice (*n* = 4) were scanned without preceding injection with luciferin to confirm absence of signal from the plasmid itself.

### Quantitative ultramorphology

To detect the ultramorphologic changes, the kidneys were cut into 1 mm slices and every second slice randomly chosen. From these slices two 1.5 mm x 1.5 mm blocks were sampled from the cortical area. These were embedded in Epon, contrasted, and 2- to 3-μm sections were cut until at least one glomerulus appeared. 50-nm sections were cut using an ultramicrotome (Leica Ultracut UCT). Using a Phillips CM10 electron microscope with a Quemesa, Olympus camera and analysis software ITEM version 3.1 the glomeruli and proximal tubuli regions were photographed. At ×10,000 original magnification the GBM and TBM thicknesses were measured where they intersected with test lines, and the harmonic means were calculated [28].

### Epo ELISA

In order to verify the production of Epo in the mice and to quantify expression, serum Epo levels were measured using the Quantikine mouse erythropoietin kit (Cat.no: MEP00B, R&D Systems, Minneapolis, MN, USA). This assay utilises the quantitative sandwich enzyme immunoassay technique, in which an antibody specific for Epo has been pre-coated onto a microplate. The optical density was measured using a microplate reader at 450 nm and the result was calculated using a four parameter logistic curve fit. The minimum detectable dose for this assay was 18.0 pg/mL.

### Urinary albumin excretion

To measure changes in kidney function after Epo treatment, the albumin concentration in 24-hours urine samples was determined using a commercial ELISA kit specific for mouse albumin (Cat.no: E90-134, Bethyl Laboratories, Montgomery, TX, USA). The urine was diluted 1:100 to 1:400 and all samples were run in duplicates.

### Total collagen content

Renal content of collagen was estimated by determining the quantity of hydroxyproline as described [29]. Kidneys from all treatment groups were defatted in chloroform:methanol 2:1 stirring for 22 hours at 4°C. The tissue was weighed and 10 mg dried and pulverised tissue was dissolved in 200 μl 6 M HCl and hydrolyzed at 118°C for 18 hours. The content of hydroxyproline was measured in the supernatant and total collagen content was calculated from total hydroxyproline using 7.46 as correction factor [29].

### Quantitative RT-PCR

At the end of the study period quantitative RT-PCR for Epo was performed on kidneys (*n* = 11) and liver (*n* = 11), as well as from other relevant organs (limb-muscle, spleen, heart, thymus, lung, brain, and testis) from a subpopulation of Tg mice (Tg Epo 1.25 μg/mouse (*n* = 3)). As markers for renal fibrosis the RNA expression of collagen and fibronectin were analysed in kidney tissue with quantitative RT-PCR. In short, total RNA from one quarter of a kidney or 5 mm x 5 mm liver sample was isolated using a homogeniser and Trizol reagent (Invitrogen, Carlsbad, CA, USA) according to the manufactures’ protocol. To make cDNA, 0.5 μg of total RNA was reverse-transcribed by Superscript III using random hexamer primers as suggested by the supplier (Invitrogen, Carlsbad, CA, USA). Real-time polymerase chain reaction (RT-PCR) was carried out and analysed by iCycler apparatus and software (Bio-Rad, Hercules, CA, USA). The primer pairs used in the study were: Epo (forward: 5’-GAGGCAGAAAATGTCACGATG-3’, reverse: 5’-CTTCCACCTCCATTCTTTTCC-3’); collagen α1(III) (forward: 5’-TGGTTTCTTCTCACCCTTCTTC-3’, reverse: 5’-TGCATCCCAATTCATCTACGT-3’); collagen α1/α2(IV) (forward: 5’-TCCTGCTTTCCGCTCTGC-3’, reverse: 5’-GAAAGGCCTTGGCTGTCACT-3’); collagen α3/α4(IV) (forward: 5’-ACGGTGTGTTCCTTGTCTCC-3’, reverse: 5’-TTCTCTTCACGGTGTGCTTG-3’); fibronectin (forward: 5’-GTGGCTGCCTTCAACTTCTC-3’, reverse: 5’-GTGGGTTGCAAACCTTCAAT-3’); and mouse housekeeping glyceraldehydes 3-phosphat dehydrogenase (GAPDH) (forward: 5’-ATGTTCCAGTATGACTCCACTCACG-3’, reverse: 5’-GAAGACACCAGTAGACTCCACGACA-3’). All the reactions were performed with SYBR green (Bio-Rad, Hercules, CA, USA) under the same conditions as follows: 95°C for 5 minutes followed by 45 cycles of 95°C/30 seconds (denaturation), 60°C/1 minute (annealing), 72°C/45 seconds (extension), and 72°C/5 minutes (final extension). A standard curve (1:5, 1:25, 1:125, 1:625) was mixed from all the cDNA samples and included in each PCR reaction. The ratio of the target gene to the housekeeping gene was calculated and all the results were located within the standards.

### Western blot

Proteins from renal tissue were extracted with lysis buffer and analysed by Western blotting to quantify the level of fibronectin accumulation in the kidneys. Twenty μg of denatured proteins were loaded in equalised amounts, electrophoresed under reducing conditions on a 4–12% criterion TX Bis-Tris gel (Bio-Rad, Hercules, CA, USA), and transferred onto a polyvinylidene fluoride (PVDF) membrane for the detection of fibronectin. The membranes were blocked in 1% Tween dissolved in Tris-buffered saline and incubated at 4°C over night with fibronectin rabbit polyclonal antibody (1:5000, Cat.no: A0245, Dako A/S, Glostrup, Denmark). The western signal was developed with horseradish peroxidase-tagged secondary antibody and luminal-based enhanced chemiluminescent substrate (Thermo Scientific, Rockford, IL, USA). The total amount of protein loaded was measured using the Stain Free Technology [30] and Image Lab (v4.0.1, Bio-Rad, Hercules, CA, USA). Fibronectin was quantified relative to the total amount of protein loaded for each specific sample.

### Statistical methods

All data are presented as mean ± standard deviation (SD). The statistical analyses were performed using Sigma Plot version 11.0. Kolmogorov-Smirnov test with Lillefors’ correction was used to test all data for normality. When more than two groups were compared, ANOVA was applied utilising the Holm-Sidak test for adjustment of multiple comparisons. In the presence of unequal variance or if the data did not follow a normal distribution, ANOVA on Ranks was used, followed by Dunn’s or Newman-Keuls test. A 2-sided Fisher’s exact test was used to test for significant differences in the association between two different groups. Pairwise comparisons were evaluated using the Student’s t-test and all values were considered significant at *P* < 0.05.

## Results

### Effect of gene therapy

**Fig 1:**
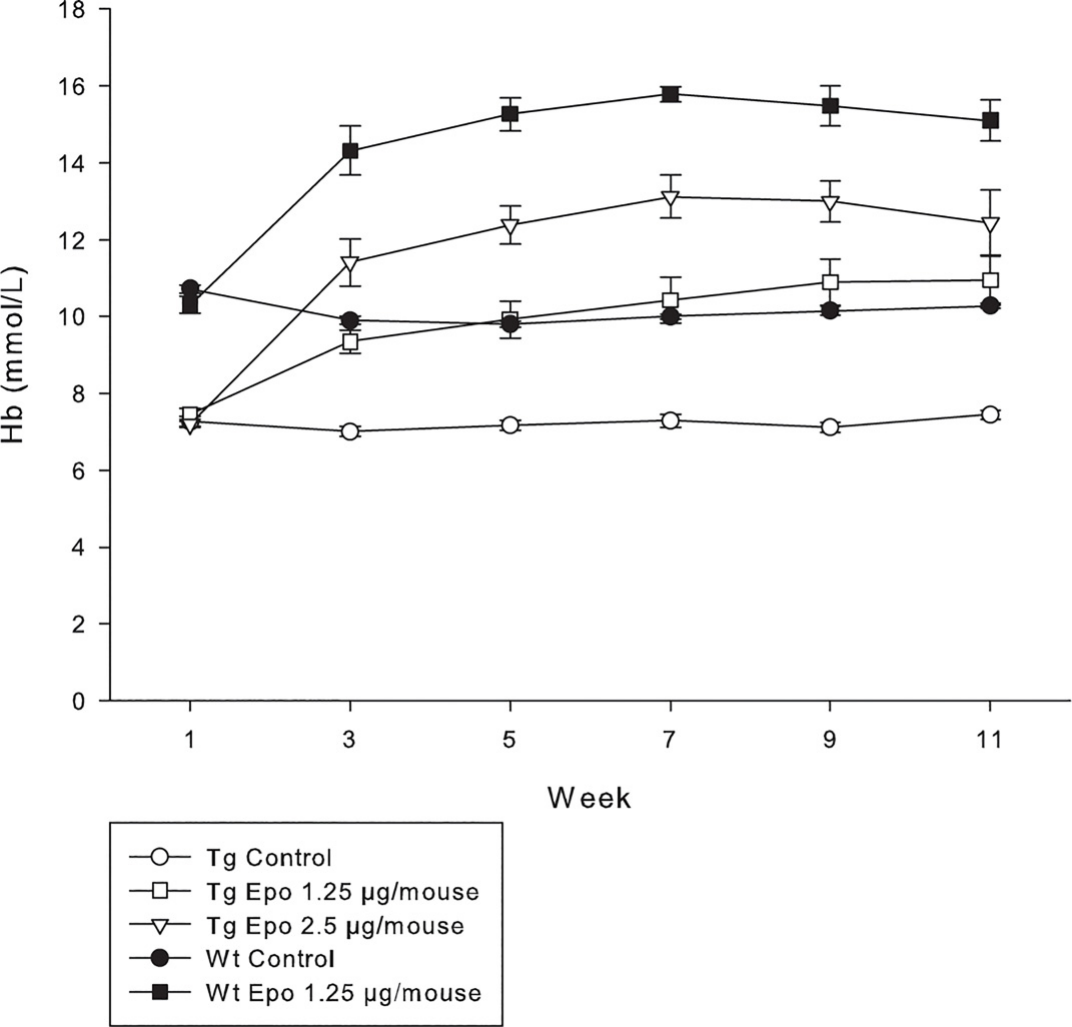
Haemoglobin measurements. Haemoglobin (Hb) is measured in the blood from the mice. Tg mice (open symbols) are anaemic compared to Wt mice (solid symbols), but the haemoglobin levels are restored in Tg mice 2 weeks after injection.

The haemoglobin level in 2-month-old Tg mice was 7.3 ± 0.5 mmol/L compared to Wt mice 10.7 ± 0.3 mmol/L (Fig 1), which is in accordance with previous described results [31]. We evaluated the time course of the biologic effect of Epo expression by weekly measurements of haemoglobin from small blood samples (Fig 1). The haemoglobin levels were unchanged in both the Tg Control (*n* = 13–15) and Wt Control (*n* = 11–15) during the entire treatment period. However, two weeks after injection with Epo plasmid the haemoglobin levels in Tg Epo 1.25 μg/mouse (*n* = 11–13) and Tg Epo 2.5 μg/mouse (*n* = 11–15) mice raised from 7.5 ± 0.6 mmol/L and 7.2 ± 0.3 mmol/L to 9.4 ± 1.2 mmol/L and 11.4 ± 2.2 mmol/L, respectively. The haemoglobin levels in Wt Epo 1.25 μg/mouse (*n =* 11) were increased from 10.7 ± 0.3 mmol/L to 15.5 ± 0.5 mmol/L. Haemoglobin levels were normalised in Tg mice already at the lowest dose of Epo and the effect was sustained during the entire observation period (*P* < 0.001, week 1 vs. week 11). A similar pattern was observed in Wt Epo 1.25 μg/mouse (*P* < 0.001, week 1 vs. week 11). These data indicate that continuous expression and translation of functional Epo protein under the control of the ubiquitin promotor can be achieved for more than 11 weeks after a single hydrodynamic injection and that long-lasting normalisation of the haemoglobin level is attained.

### Animal health and body weight

Transgenic RenTGF-β1 and Wt mice were injected with 1.25 μg/mouse Epo plasmid, 2.5 μg/mouse Epo plasmid, or 5 μg/mouse Control plasmid contained in a volume of 8% of their bodyweights. Immediately after the hydrodynamic gene transfer procedure, the bodyweights of the mice were elevated in average 2.0 ± 0.92 g corresponding to the injected volume (*n* = 69), but were normalised 24 hours later (S1 Fig). The mice were weighed weekly and they all gained weight during the whole 11 weeks period (Table 1). Behaviour and grooming activity were similar in the 5 groups throughout the study period, which indicate that the injection itself did not affect the well-being of the mice. Dissected organs showed no apparent signs of necrosis or disruption (S2 Fig). Not all animals completed the 11 week protocol (Table 1), but died between 2 and 7 weeks after injection. The number of dead mice was neither dependent on the mouse type or the treatment dose (*P* > 0.43, 2-sided Fisher’s exact test).

**Table 1:** Mouse weights and numbers of mice in each group during the study period.

	Transgenic (Tg)			Wildtype (Wt)	
	Control	Epo 1.25 μg/mouse	Epo 2.5 μg/mouse	Control	Epo 1.25 μg/mouse
**Week 1**	20.3 (±4.3)*n* = 15	21.5 (±2.4)*n* = 13	22.0 (±3.9)*n* = 15	24.9 (±3.1)*n* = 15	27.3 (±3.1)*n* = 11
**Week 5**	23.8 (±3.7)*n* = 14	22.9 (±2.6)*n* = 13	23.5 (±4.9)*n* = 14	28.2 (±3.5)*n* = 15	30.4 (±2.3)*n* = 11
**Week 10**	25.9 (±3.8)*n* = 13	24.0 (±2.9)*n* = 11	27.4 (±2.9)*n* = 11	31.3 (±1.5)*n* = 11	32.4 (±2.5)*n* = 11

Values are means ± SD.

### Plasmids are expressed in the liver region and Epo delivered systemically

**Fig 2:**
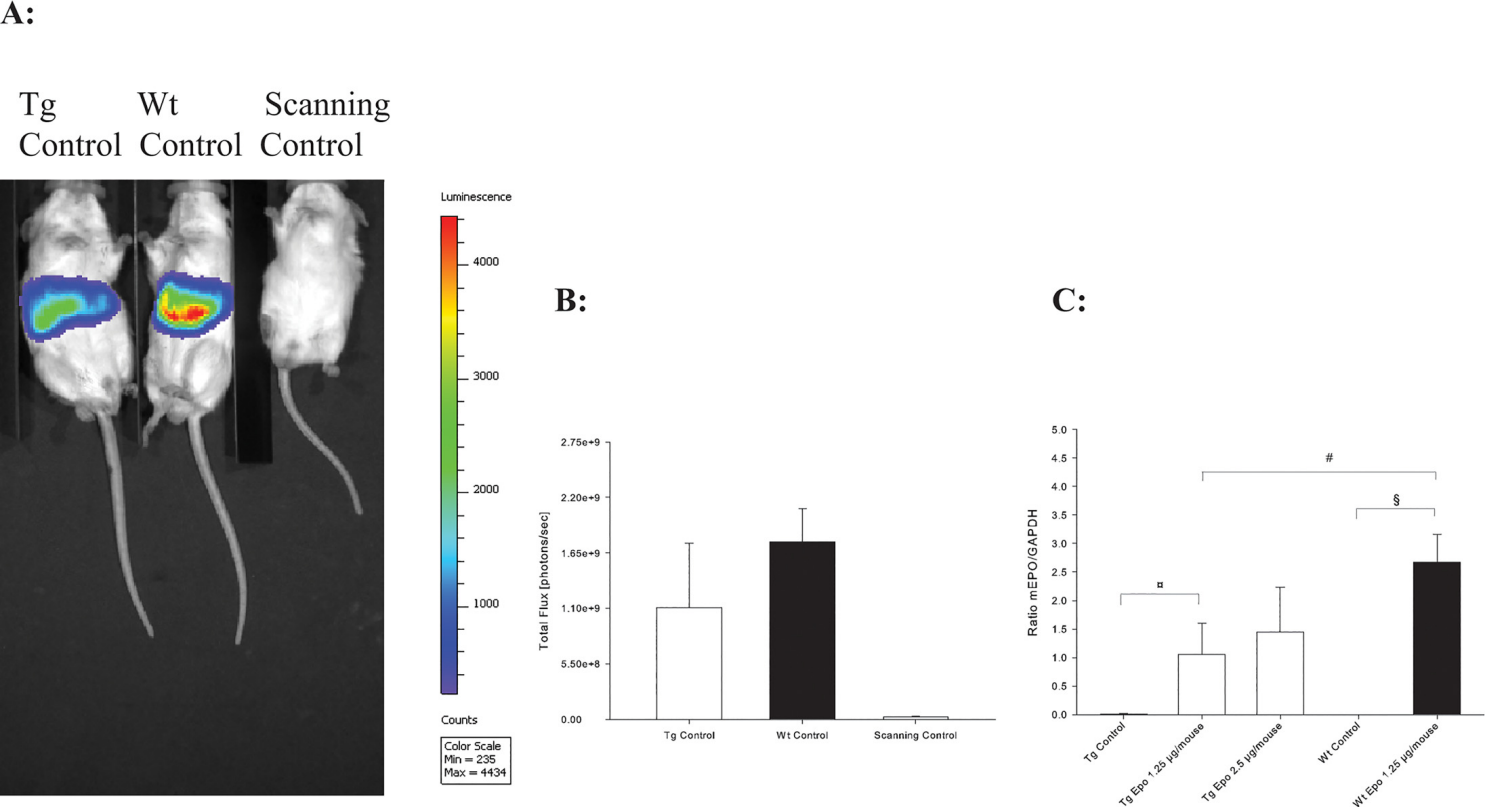
Evaluation of plasmid expression site and level. **A:** Plasmid signal day 8 after injection in Tg Control (*n =* 8) and Wt Control (*n =* 5) corresponding to the location of the liver. Scanning Control mice without preceding injection with luciferin are scanned simultaneously (*n =* 4). **B:** Regions of interest marks the signal and is measured as total flux (photons/second) (± SD). There is a tendency to higher plasmid expression in Wt Control mice (*n =* 5) than Tg Control mice (*n =* 8) (*P* = 0.13). **C:** Expression of Epo mRNAfrom the liver at termination of the experiment. Both Tg (*n =* 11) and Wt (*n =* 11) mice treated with Epo express significant more plasmid from the liver compared to their Controls (^*¤*,*§*^ *P* < 0.001, ± SD). Significant difference between Tg Epo 1.25 μg/mouse (*n =* 11) and Wt Epo 1.25 μg/mouse (*n =* 11) (^*#*^ *P* < 0.001, ± SD) are found.

To confirm plasmid expression in the liver, we injected a group of Tg and Wt littermates with Control plasmid following the same procedure as described above. After 8 days the mice were injected intraperitoneal with luciferin (Tg *n* = 8, Wt *n* = 5). The mice were visualised and light emission was measured. As expected, the light emission was observed in mice treated with Control plasmid corresponding to the location of the liver. No light emission was seen in the Scanning Control without injection of luciferin (Fig 2A and 2B). Wildtype mice had a tendency to express more luciferase than Tg mice, however, this was not statistically significant (*P* = 0.13).

As expected, no expression of Epo mRNA in the liver was observed in Tg Control (*n =* 13) and Wt Control (*n* = 11) (Fig 2C). Expression of Epo mRNA in liver tissue from both Tg (*n* = 11) and Wt (*n* = 11) mice receiving Epo rose significantly compared to the respectively Controls regardless of the Epo plasmid concentration (^*¤*, *§*^ *P* < 0.001, ± SD). This indicates that the effect of hydrodynamic gene transfer was sustained for more than 11 weeks. The Wt Epo 1.25 μg/mouse (*n* = 11) had a higher level of expression of plasmid than Tg Epo 1.25 μg/mouse (*n* = 11) (^#^ *P* < 0.001), which is in accordance with the slightly increased luciferase signal in Wt mice reported above. The expression of Epo was not significantly different between Tg Epo 1.25 μg/mouse and Tg Epo 2.5 μg/mouse. Epo mRNA expression was confined only to the liver or the kidneys (endogenous production) in the mice that received Epo plasmid injection. No Epo mRNA expression was observed in other tested organs (heart, thymus, lung, brain, limb-muscle, testis, and spleen) (S3 Fig).

**Fig 3:**
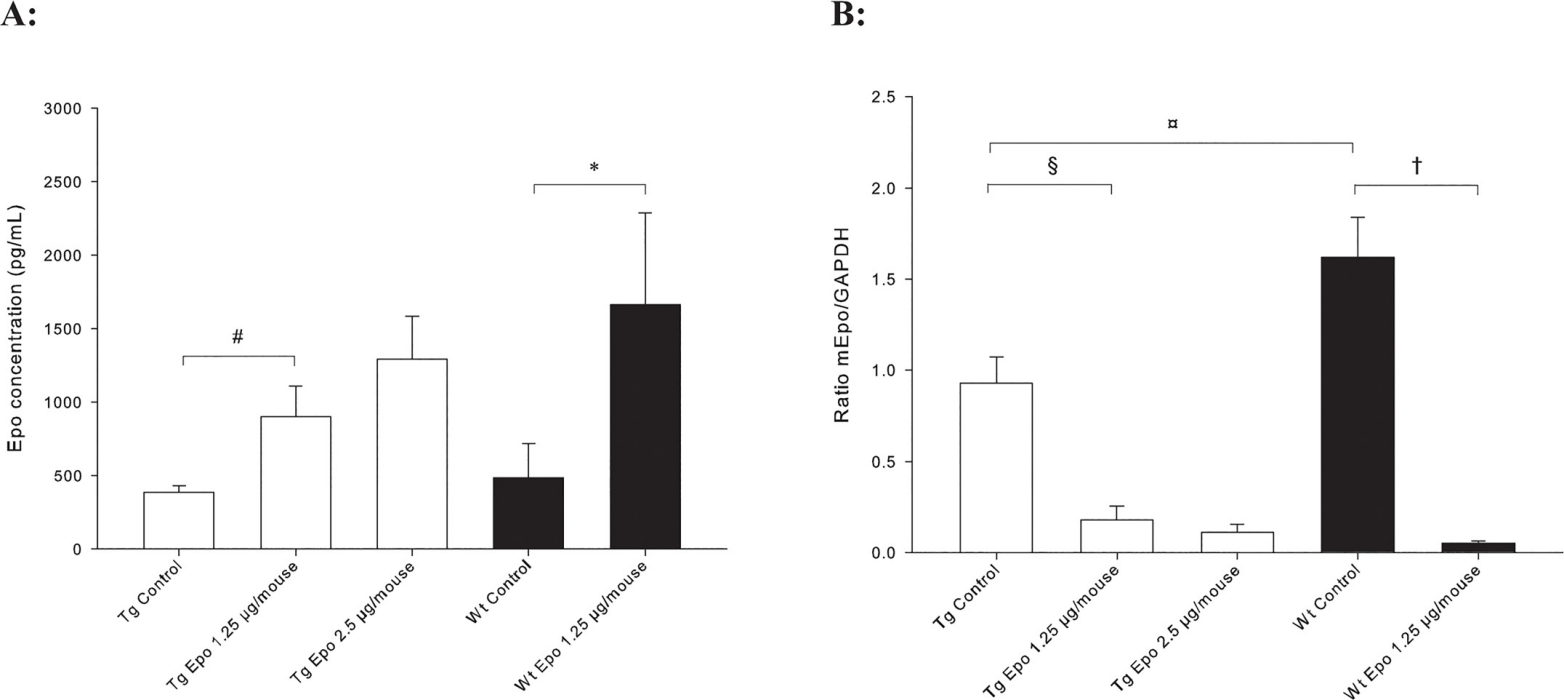
Epo expression. **A:** Epo protein is significant higher in serum samples after 11 weeks for both Tg (*n =* 10) and Wt (*n =* 6) mice receiving Epo 1.25 μg/mouse compared to their Controls (*n =* 11 and *n =* 9, respectively) (^*#*^ *P* = 0.004 and *P < 0.001, ± SD). **B:** Epo mRNA expression from kidneys. Significant decreased expression is seen in Tg Control (*n =* 13) compared to Wt Control (*n =* 11) (^*¤*^ *P* = 0.025, ± SD). Treatment with Epo plasmid decreases endogenous Epo expression in both Tg and Wt mice (^*§*,*†*^ *P* < 0.001, ± SD).

The serum concentration of Epo was measured at the completion of the experiment. As expected, both Tg mice (*n* = 10) and Wt mice (*n* = 6) that received Epo 1.25 μg/mouse showed significant higher Epo concentrations in serum than their control littermates (Fig 3A, ^*#*^ *P* = 0.004 and *P < 0.001). There were no significant differences between Tg mice receiving Epo 1.25 μg/mouse (*n* = 10) and Epo 2.5 μg/mouse (*n* = 8) or between Tg Control (*n* = 11) and Wt Control (*n* = 9), respectively. It was unexpected that Tg Controls had almost the same level of Epo as Wt Controls. However, this is in concordance with the observation that Epo mRNA expression is present in the Tg Control mice (*n* = 13), although at a significant lower level compared to the Wt Control mice (*n* = 11) (^¤^P = 0.025) (Fig 3B). Treatment with Epo plasmid reduced the Epo expression in the kidneys in Tg Epo 1.25 μg/mouse (*n* = 11), Tg Epo 2.5 μg/mouse (*n* = 11), and Wt Epo 1.25 μg/mouse (*n* = 11) compared to their respective Control group (Fig 3B), indicating a negative feedback by the exogenous produced Epo from the liver. The presence of Epo in the circulation and expression of Epo mRNA might indicate that the Epo produced in the fibrotic kidneys in Tg mice is non-functional either because of post-translational modifications or other causes. Inflammatory effector molecules may affect Epo function. However, previous studies performed in this transgenic model, we find inflammatory cells only at the later stages of kidney fibrosis development [32].

### Epo administration does not attenuate TGF-β1-mediated total collagen expression in the kidneys

Transgenic mice had elevated collagen depositions in the kidneys compared to Wt mice (*P < 0.001, Tg Control *n* = 8 v.s. Wt Control *n* = 5) (Table 2). This is in accordance with our previously results [31,33]. Treatment with Epo 1.25 μg/mouse was without impact on the collagen content in both Tg and Wt mice. There was no significant difference between Tg Epo 1.25 μg/mouse (*n* = 8) and Tg Epo 2.5 μg/mouse (*n* = 8) (Table 2).

**Table 2:** Morphologic and functional characteristics on kidneys from transgenic and wildtype mice receiving Epo plasmid.

		Transgenic (Tg)			Wildtype (Wt)	
	*n*	Control	Epo 1.25 μg/mouse	Epo 2.5 μg/mouse	Control	Epo 1.25 μg/mouse
**Total collagen (μg/mg tissue)**	5–8	30(27.9–32.2)	31 (28.5–34.7)	29(25.7–31.2)	21*(17.7–23.4)	19(15.8–22.3)
**Albumin excretion (μg albumin/24 hours)**	3–6	56(25–87)	-	148(52–242)	-	-
**GBM (nm)**	5–8	270 ±14.4	264 ±39.9	278 ±24.3	179 ±7.6*	166 ±17.4
**TBM (nm)**	5–8	214 ±32.0	238 ±33.9	212 ±26.2	172 ±18.7^#^	187 ±14.3

Values are means ± SD or 95% CI. GBM, glomerular basement membrane; TBM, tubular basement membrane.

*P < 0.001 vs. Tg Control and

^*#*^ *P* = 0.01 vs. Tg Control.

### Functional data

Albumin excretion in 24-hours urine was measured in Tg Control (*n* = 3) and Tg Epo 2.5 μg/mouse (*n* = 6) after the treatment period (Table 2). There was no significant difference between the two groups, which suggests that Epo treatment does not have any effect on the functional part of the kidney. Unfortunately it was not possible to collect 24-hours urine from more mice to increase the number of animals in each group due to unexpected technical difficulties.

### Epo administration does not attenuate TGF-β1-mediated thickening of GBM

**Fig 4:**
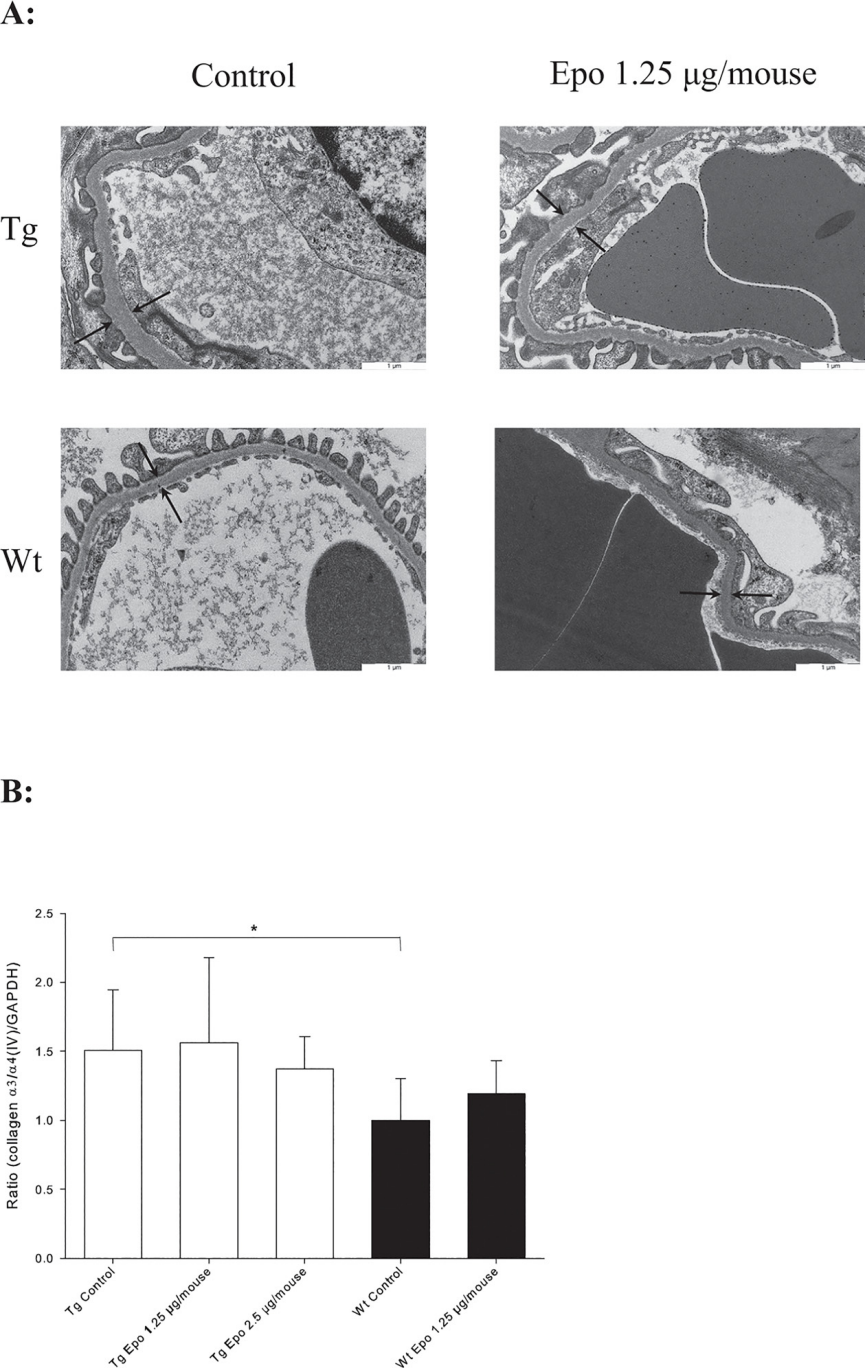
Evaluation of the glomerular basement membrane (GBM). **A:** Thickening of GBM evaluated by electron microscopy. Arrows indicate GBM thickness. **B:** Collagen α3/α4(IV) mRNA expression is increased in Tg Control (*n =* 8) compared to Wt Control mice (*n =* 5) (*P < 0.01, ± SD), but treatment with Epo has no effect in either type.

Electron microscopy measurements were used to estimate the thickness of the GBM. As shown previously [33,34], the GBM was significant thicker in the Tg mice (*n* = 8) compared to the Wt mice (*n* = 5) (*P < 0.001) (Table 2 and Fig 4A). Administration of Epo plasmids did not change the thickness of GBM irrespective of the genotype. These results were paralleled by biochemical data. A central component of the mature GBM is collagen α3/α4/α5(IV) [35]. We found increased expression of collagen α3/α4(IV) mRNA as measured by RT-PCR comparing Tg Control (*n* = 8) with Wt Control (*n* = 5) (*P < 0.01) (Fig 4B), but Epo was without effect on collagen α3/α4(IV) mRNA expression regardless of the mouse type. There was no difference between Tg mice treated with Epo 1.25 μg/mouse and Epo 2.5 μg/mouse.

### Epo administration does not attenuate TGF-β1-mediated thickening of TBM or interstitial fibrosis

**Fig 5:**
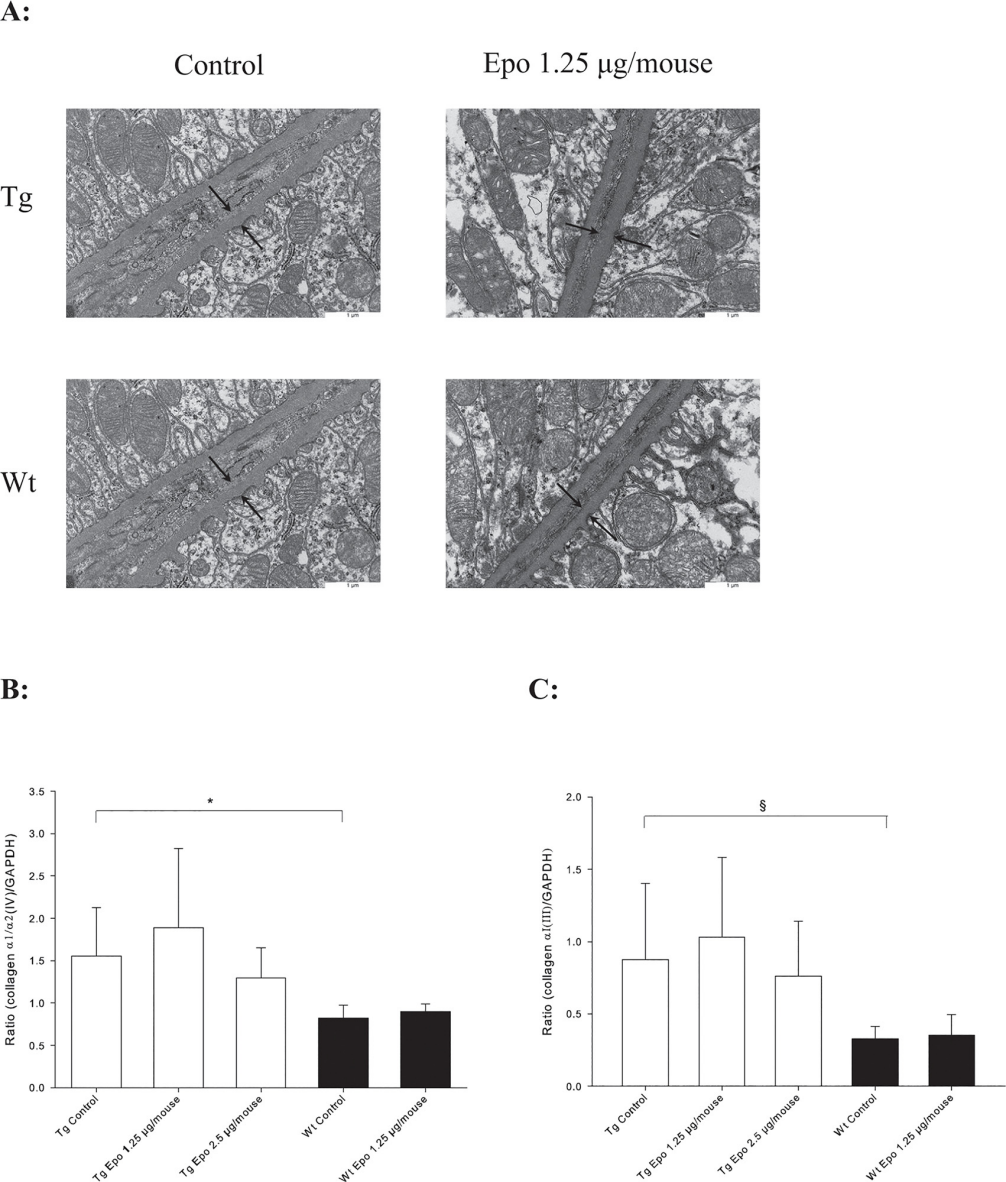
Evaluation of the tubular basement membrane (TBM). **A:** Thickening of TBM evaluated by electron microscopy. Arrows indicate TBM thickness. **B:** Collagen α1/α2(IV) mRNA expression is increased in Tg Control (*n =* 8) compared to Wt Control mice (*n =* 5) (*P < 0.001, ± SD), but treatment with Epo has no effect in either type. C: Collagen αI(III) mRNA expression is increased in Tg (*n =* 8) compared to Wt mice (*n =* 5) (^*§*^ *P* < 0.01, ± SD), but treatment with Epo has no effect in either type.

Thickening of the tubular basement membrane (TBM) was estimated by electron microscopy and evaluated by expression of collagen α1/α2(IV), which is found in excessive amounts in the TBM network. Transgenic Control (*n* = 8) showed thickening of TBM compared with Wt Control (*n* = 5) (^*#*^ *P* = 0.01) (Table 2 and Fig 5A). This observation was confirmed by increased collagen α1/α2(IV) expression in the Tg Control (*n* = 13) compared with Wt Control (*n* = 15) (*P = 0.001) (Fig 5B). Treatment with Epo plasmid did not change TBM thickness in either Tg or Wt mice and no effect was seen in collagen α1/α2(IV) expression (Table 2 and Fig 5A and 5B).

Tubulointerstitial fibrosis is furthermore characterised by accumulation of extracellular matrix in the interstitial space with primarily increased expression of interstitial fibrillar collagen α1(III) [36]. Transgenic Control mice (*n* = 13) expressed more collagen α1(III) mRNA than Wt Control mice (*n* = 15) (^*§*^ *P* < 0.01), but no effects of treatment with Epo were seen in either the Tg or Wt mice (Fig 5C).

### Fibronectin content

**Fig 6:**
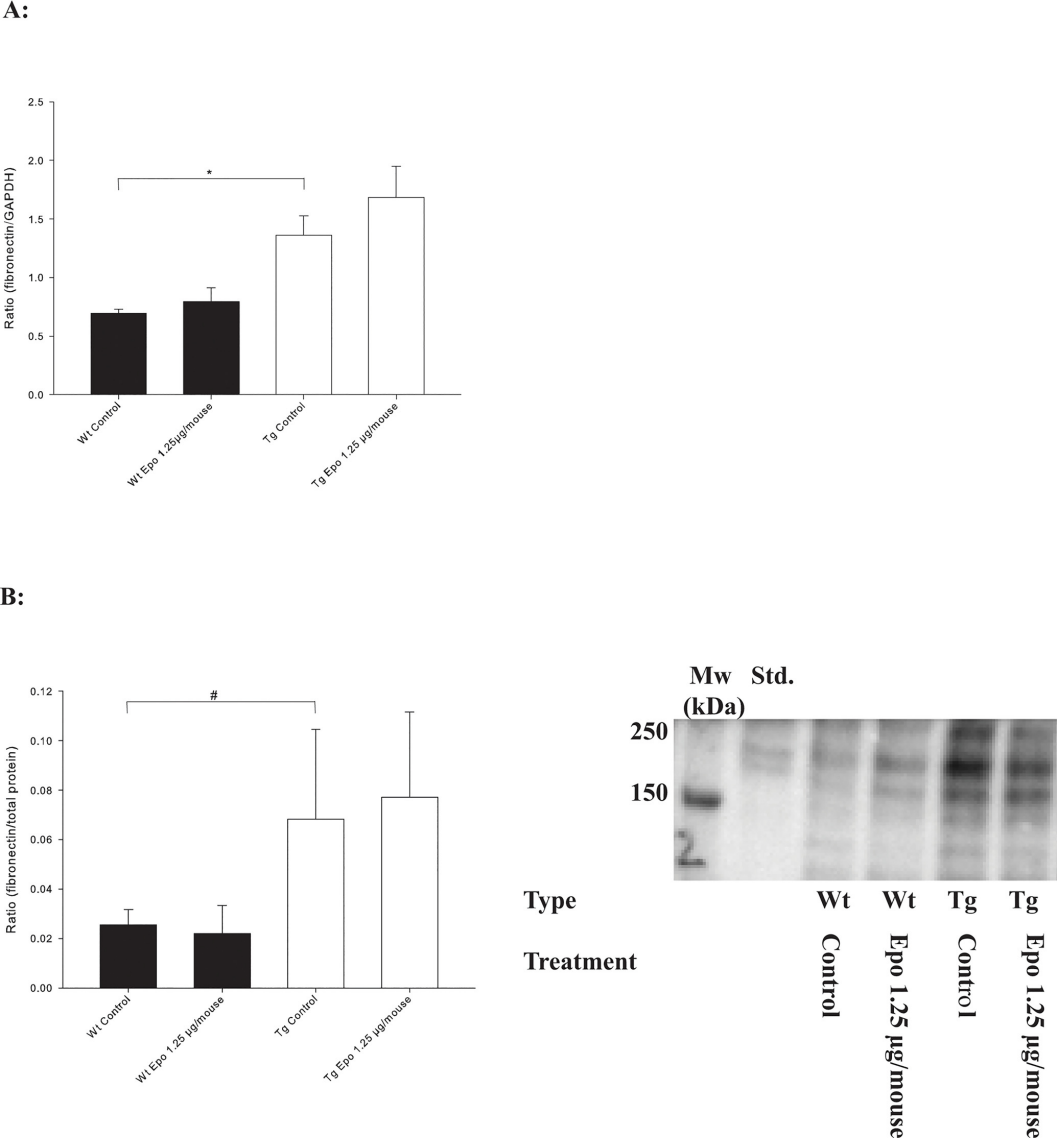
Expression and content of fibronectin in kidneys. **A:** Transgenic Control mice (*n =* 13) express more fibronectin mRNA in total kidney tissue compared with Wt Control mice (*n =* 11) (*P < 0.001, ± SD), but treatment with Epo has no effect in either type (*n =* 11 for both types). **B:** Total fibronectin protein is present in increased amounts in transgenic mice (^*#*^ *P* < 0.001, ± SD), but treatment with Epo has no effect. A representative section of the western blot is shown.

Fibronectin mRNA expression was significantly elevated in Tg Control mice (*n* = 13) compared to Wt Control mice (*n* = 11) (*P < 0.001) (Fig 6A). Treatment with Epo had no effect on fibronectin content in either the Tg or Wt mice. These results were paralleled by changes in the fibronectin quantity in total kidney tissue as estimated by Western blotting. The fibronectin content was statistically increased in Tg Control mice (*n* = 11) compared with Wt Control mice (*n* = 6) (^*#*^ *P* < 0.001), whereas treatment with Epo had no effect on the fibronectin content in either the Tg Epo 1.25 μg/mouse (*n* = 8) or Wt Epo 1.25 μg/mouse (*n* = 5) (Fig 6B).

## Discussion

The major findings of the present study are 1) long term normalisation of anaemia is possible with a single hydrodynamic injection of Epo plasmid 2) long term Epo secretion does not affect kidney morphology or the content of extracellular matrix components in a transgenic mouse model of chronic kidney disease, and 3) long term Epo secretion has no adverse effect on kidneys in normal Wt mice.

Prolonged effect of Epo expression was effective in the present mouse model since a sustained elevation in the haemoglobin level was maintained during the 11 week study period after a single hydrodynamic injection. We noticed higher mRNA Epo expression, higher physiologic response in haemoglobin levels, and a tendency to more bioluminescence signal in Wt mice treated with the similar doses, suggesting a difference in plasmid transfection of the liver cells. This could be because the Tg mice are anaemic and hence have different blood compositions and blood viscosity, which might impact the hydrodynamic transfections. The haemoglobin levels in the Tg mice were however normalised already 2 weeks after injection with the lowest dose of Epo plasmids.

The exact mechanism for the gene transfer is not fully elucidated, but it is believed that the rapid injection of the large volume might cause a transient right-sided congestive heart failure with backflow to the liver vessels and subsequent plasmid transfection of liver cells [21]. The relative slow turnover of hepatocytes in adult animals could explain the long-term plasmid expression [37].

All the animals survived the hydrodynamic procedure and the overall health of the animals throughout the study period appeared unaffected. The hydrodynamic gene transfer method for expressing exogenous protein from the liver cells has previously been used with success to deliver Epo plasmid, growth hormone, or Human Factor IX in vivo in rats and mice [21,22,38]. We cannot exclude a slight liver damage when injecting such a large volume [23], but the regenerative capabilities of the liver make this damage transient and we find no signs of liver damage after the study period.

Clinical studies suggest that treatment with recombinant human Epo (rhEpo) in pre-dialysis patients can be advantageous because it diminishes the risk of cardiovascular disease and reduce the mortality [39,40]. The studies further indicate that rhEpo treatment slows the loss of renal function. Limitations of the studies, however, are the advanced stage of the renal failure and the short observation period [10]. In the current study we use a mouse model for chronic kidney disease that slowly develops glomerular sclerosis and tubulointerstitiel fibrosis beginning when the mice are 2-month-old. This is also the age at which the treatment with Epo plasmid is initiated and the expression of Epo is sustained throughout the period the fibrosis in the kidneys develops into a manifest stage. The fact that the kidneys are exposed to Epo before the glomerular- and tubulointerstitiel fibrosis is obvious, allows for a correct assessment of the potential beneficial effects of Epo.

Interestingly, the biochemical, functional, and ultramophologic changes were similar in Tg mice treated with Epo or Control. This indicates that correction of anaemia to normal levels or prolonged exposure to Epo does not affect the progression of TGF-β1-induced chronic kidney disease. Treatment with rhEpo enhances the functional recovery in rats with acute renal failure [41]. Most renal cells are found to express receptors for Epo [42] which could indicate that Epo exerts paracrine effects in addition to the regulation of the erythropoietic system. In a study by Nemeto *et al* treatment with high dose Epo restored haemoglobin levels and decreased mortality in rats with ischemic acute renal failure but had no effect on serum creatinine levels [43] and other groups have found similar non-beneficial effects of Epo treatment [10,13,44]. It has been speculated that these inconsistent effects of a hyper-physiologic dose of Epo cause renal vasoconstriction and reduce the beneficial effect on glomerular filtration [42]. In the present study we cannot exclude that the supra-physiologic concentrations of haemoglobin lead to hypertension, which may outweigh the potentially positive effects of Epo. Therefore this experimental study cannot adequately rule out a direct effect of Epo on renal cells or the consequences of correction of anaemia on the progression of TGF-β1-induced chronic kidney disease. Finally, only male mice are investigated and we cannot rule out the possibility of a beneficial effect in females. Further studies including a blood pressure measurement and both sexes are needed. Furthermore, it could be interesting to investigate why the Epo produced in the kidneys in the Tg mice presumably do not have a physiological action.

In conclusion, this study shows that hydrodynamic gene therapy can be used to treat anaemia in transgenic mice. However, the present biochemical, functional, and morphological findings suggest that long-term Epo expression has no beneficial effects on TGF-β1-induced chronic kidney disease in the time frame and dose provided in this study. On the other hand Epo does not worsen any of the investigated parameters in transgenic mice and Epo seems not to be harmful for kidney morphology and extra cellular matrix turnover in normal mice.

Further studies need to be performed to confirm that Epo treatment has no adverse effects on kidneys.

## Supporting Information

S1 Fig Mouse weights measured the day before injection (Weight -1), immediately after injection (Weight 0) and 24 hours after injection (Weight +1). Values are means ± SD.
(DOCX)

S2 Fig Representative Hematoxylin eosin stainings of liver (A), kidney (B), and spleen (C) sections from Tg or Wt mice treated with Control or Epo 1.25 μg/mouse. (JPG)

S3 Fig Epo mRNA expression in Tg mice injected with Epo 1.25 μg/mouse in relevant organs (*n* = 3). Epo expression is only observed from liver and kidneys.
(EPS)
