# Supplementary material for: Restoration of Haemoglobin Level Using Hydrodynamic Gene Therapy with Erythropoietin Does Not Alleviate the Disease Progression in an Anaemic Mouse Model for TGFβ1-Induced Chronic Kidney Disease
Source: PLoS One. 2015 Jun 5;10(6):e0128367. doi: 10.1371/journal.pone.0128367 (PMC4457485; doi:10.1371/journal.pone.0128367)
Supplement: S1 Fig — Values are means ± SD. (DOCX) [file pone.0128367.s001.docx]

|  | Transgenic (Tg) | | |  | Wildtype (Wt) | |
| --- | --- | --- | --- | --- | --- | --- |
|  | Control  *n* = 15 | Epo 1.25 µg/mouse  *n* = 13 | Epo 2.5 µg/mouse  *n* = 15 |  | Control  *n* = 15 | Epo 1.25 µg/mouse  *n* = 11 |
| Weight -1 | 20.6 (±3.6) | 21.7 (±3.1) | 22.3 (±1.9) |  | 25.8 (±1.5) | 27.1 (±2.2) |
| Weight 0 | 22.4 (±1.6) | 22.9 (±2.4) | 25.7 (±2.3) |  | 28.0 (±2.1) | 29.7 (±2.9) |
| Weight +1 | 20.3 (±4.3) | 21.5 (±2.4) | 22.0 (±3.9) |  | 24.9 (±3.1) | 27.3 (±3.1) |
