# Supplementary figures and images for: Restoration of Haemoglobin Level Using Hydrodynamic Gene Therapy with Erythropoietin Does Not Alleviate the Disease Progression in an Anaemic Mouse Model for TGFβ1-Induced Chronic Kidney Disease
Source: PLoS One. 2015 Jun 5;10(6):e0128367. doi: 10.1371/journal.pone.0128367 (PMC4457485; doi:10.1371/journal.pone.0128367)

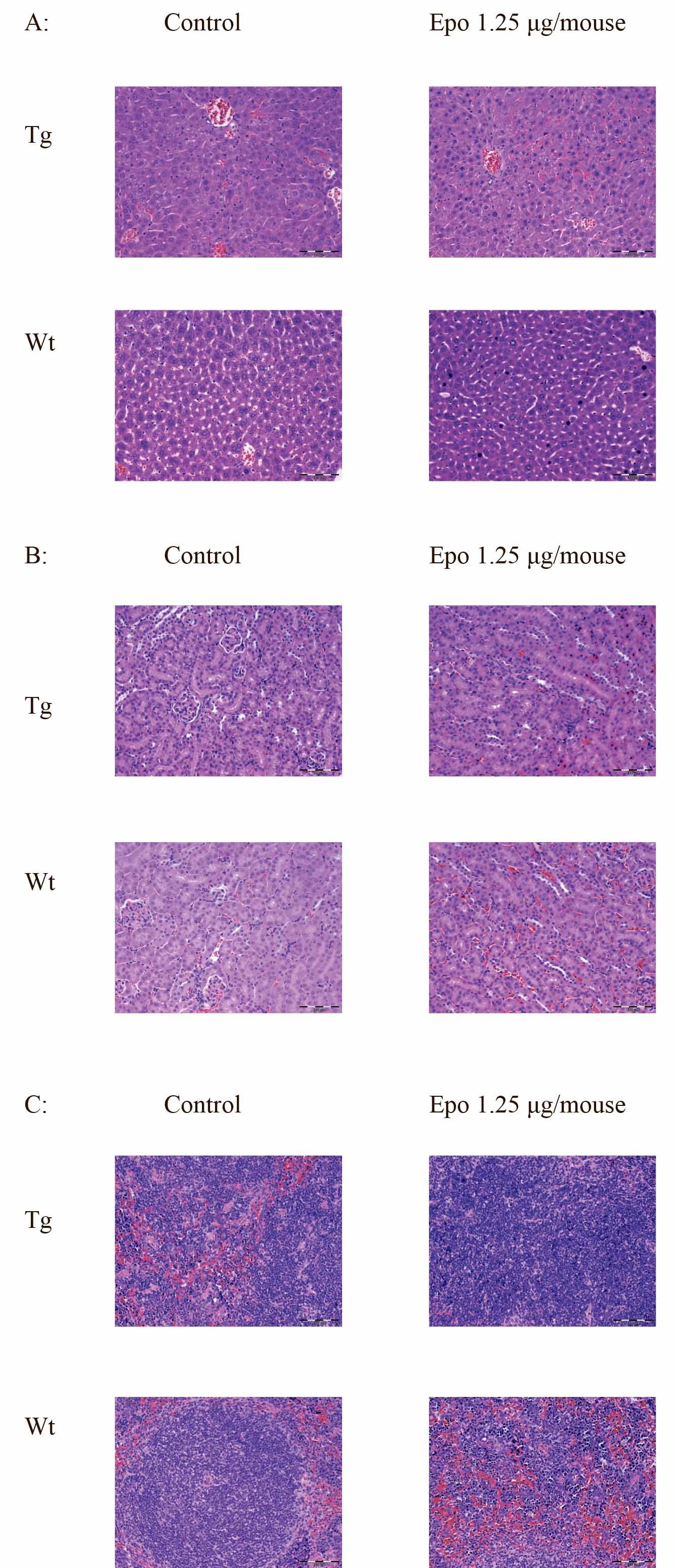

Supplement: S2 Fig — (JPG) [file pone.0128367.s002.jpg]
